# Restricted neural parametric modulation of emotional arousal in autism reveals a core role for the cerebellum

**DOI:** 10.1093/scan/nsag026

**Published:** 2026-04-09

**Authors:** Daniel Agostinho, Daniela Sousa, Miguel Castelo-Branco, Marco Simões

**Affiliations:** Center for Informatics and Systems of the University of Coimbra (CISUC), Faculty of Science and Technology, University of Coimbra, Coimbra, 3030-290, Portugal; Coimbra Institute for Biomedical Imaging and Translational Research (CIBIT), ICNAS, Faculty of Medicine, University of Coimbra, Coimbra, Portugal; Coimbra Institute for Biomedical Imaging and Translational Research (CIBIT), ICNAS, Faculty of Medicine, University of Coimbra, Coimbra, Portugal; Instituto Superior Miguel Torga (ISMT), Coimbra, Portugal; Coimbra Institute for Biomedical Imaging and Translational Research (CIBIT), ICNAS, Faculty of Medicine, University of Coimbra, Coimbra, Portugal; Center for Informatics and Systems of the University of Coimbra (CISUC), Faculty of Science and Technology, University of Coimbra, Coimbra, 3030-290, Portugal

**Keywords:** ASD, arousal, emotional processing, parametric modulation

## Abstract

Understanding how emotional arousal and valence are processed in autism spectrum disorder (ASD) is key to uncovering underlying affective mechanisms. Prior research has yielded mixed results, particularly regarding arousal. Using functional magnetic resonance imaging (fMRI), we examined neural responses to emotional video clips in adults with ASD (*n* = 20) and typically developing controls (*n* = 20). Stimuli were designed to elicit controlled emotional responses with standardized visual and narrative features, and parametric modulation analyses assessed neural activation as a function of self-reported arousal and valence. Behavioral ratings confirmed stimulus validity. For arousal, typically developing individuals showed widespread modulation across frontal, parietal, temporal, and subcortical regions, including the cingulate gyrus, insula, caudate, and ventral tegmental area. In contrast, ASD participants exhibited restricted modulation, primarily within the anterior cerebellum. No group differences emerged for valence, with both groups recruiting frontal and limbic regions such as the amygdala and insula. These findings highlight a dissociation between arousal and valence processing in ASD. Reduced cortical engagement and increased anterior cerebellar (including the vermis) involvement during arousal suggest compensatory mechanisms, underscoring the importance of distinguishing emotional dimensions when characterizing neural processes in autism.

## Introduction

Autism spectrum disorder (ASD) is an early-onset, lifelong neurodevelopmental condition characterized by deficits in social communication and interaction, along with restricted and repetitive patterns of behavior and interests (Hyde and Garcia-Rill 2019). A core challenge for individuals with ASD is sensory processing (Mills *et al*. 2022, Dell’Osso *et al*. 2023), which involves the reception, integration, and interpretation of sensory input to generate adaptive responses to environmental demands. Difficulties in this domain, now recognized in diagnostic criteria, affect up to 75% of individuals with ASD and are closely linked to dysregulation of autonomic and behavioral arousal (South and Rodgers 2017). When arousal responses are poorly regulated, emotional control can be compromised, giving rise to heightened stress, anxiety, and maladaptive behaviors such as self-injury, problematic behavior, and impaired decision-making (Kinsbourne 1987, Hirstein *et al*. 2001, Luke *et al*. 2012, Gotham *et al*. 2013, Buck *et al*. 2014, Kerns *et al*. 2014, Orekhova and Stroganova 2014). These sensory and emotional difficulties also place additional strain on family systems and are associated with increased risk of phobias, social dysfunction, and reduced occupational performance (Roelofs *et al*. 2009, Conner *et al*. 2013, Kashefimehr *et al*. 2018, Muskett *et al*. 2019). Understanding and improving sensory modulation and emotional regulation is therefore a critical target for enhancing adaptive functioning and overall quality of life in ASD (Guivarch *et al*. 2017, Gates *et al*. 2023, Wright *et al*. 2023).

Emotions are commonly studied through two main perspectives: Basic Emotion Theory and the Contemporary Theory of Emotion. Basic Emotion Theory proposes that discrete emotions such as fear, happiness, and anger are biologically hardwired and universally expressed (Ekman 1992, Ekman and Cordaro 2011). However, neuroimaging approaches based on this framework face important challenges, particularly the difficulty of defining truly “neutral” control stimuli, which are often ambiguous and may confound comparisons (Klein *et al*. 2015).

In contrast, Contemporary Theories of Emotion emphasize emotions as emergent states shaped by dynamic interactions of more fundamental psychological processes. Two prominent frameworks in this context are Dimensional Theory of Emotion and the Constructivist Theory of Emotion. The Dimensional Theory departs from the notion that emotions exist in distinct categories and emphasizes that emotions can be represented by core affect, which consists of two continuous dimensions: arousal (activation) and valence (pleasantness), that combine to form diverse emotional states (Russell 1980, Rubin and Talarico 2009, Hamann 2012, Fontaine 2013). This perspective better accommodates individual and contextual variability, as emotional experience depends on how the brain interprets arousal and valence signals in relation to situational cues and prior experiences (Barrett 2017, Adolphs *et al*. 2019, Cabrera 2021). The Constructivist Theory, by contrast, argues that emotions are not biologically fixed categories but are constructed by the brain through the integration of core affect with conceptual knowledge, language, and social context (Lindquist and Barrett 2012, Barrett 2017a, 2017b, Barrett and Satpute 2019). From this view, emotions emerge as flexible, context-dependent interpretations of bodily and environmental signals rather than discrete, hardwired states. Consistent with this broader contemporary framework, neuroimaging studies have identified distinct neural systems supporting arousal and valence, as well as their modulatory influence on distributed cognitive networks (Colibazzi *et al*. 2010, Citron *et al*. 2014, Bestelmeyer *et al*. 2017, Scott 2018).

This constructivist framework is particularly relevant when studying conditions like ASD, where emotional and sensory experiences often diverge from typical patterns which may reflect differences in processing arousal and valence (Uljarevic and Hamilton 2013). Rather than focusing on discrete emotions, the parametric examination of the core dimensions of arousal and valence offers a more flexible framework for analyzing emotional processing in ASD (Tseng *et al*. 2016). In the present study, we used functional magnetic resonance imaging (fMRI) to examine whether neural activity related to emotional arousal and valence differs between individuals with ASD and typically developing (TD) controls. Participants viewed immersive, first-person emotional videos from the CAAV dataset, which provides controlled, standardized stimuli for studying emotional processing (Di Crosta *et al*. 2020).

## Methods and materials

### Dataset description

#### Participants’ demographic description

We collected fMRI data from 40 adolescents and young adults (20 with ASD, 20 TD controls), aged 16–31 years and matched for age (see Table 1). Individuals with ASD were recruited from Portuguese ASD associations in Viseu and Coimbra and our volunteer database. A prior medical diagnosis was required, and ADOS-2 Module 4 (Lord *et al*. 2000) was administered by a clinical psychologist. All participants had normal intellectual functioning with a full-scale intelligence quotient estimate (FSIQ-estimate >70), assessed by the Wechsler Adult Intelligence Scale-3rd Edition (WAIS-III) short form (Velthorst *et al*. 2013). TD controls were recruited locally and underwent the same screening, except ADOS-2. The Autism Spectrum Quotient (AQ) (Baron-Cohen *et al*. 2001) was applied as an exclusion criterion (≥32). Informed consent was obtained from all participants or legal representatives in compliance with the Declaration of Helsinki. The procedure was approved by the Ethics Committee of the Faculty of Medicine, University of Coimbra.

**Table 1:** Demographic description of the ASD and TD groups, including age, Full-Scale Intelligence Quotient-estimate (FSIQ-estimate), Empathy Quotient (EQ), Autism Spectrum Quotient (AQ), and the Autism Diagnostic Observation Schedule (ADOS-2) total score.

	ASD (*n* = 20)	TD (*n* = 20)
**Age (years)**	21.30 (4.62)	22.37 (3.71)
**FSIQ-estimate**	96.05 (11.74)	115.50 (15.46)
**EQ total**	37.25 (11.56)	45.53 (7.89)
**aq total**	23.85 (4.13)	15.79 (5.62)
**ADOS-2 total**	11.20 (2.98)	

Each score is presented in terms of group average and standard error, in brackets.

#### Experimental fMRI task

**Figure 1:**
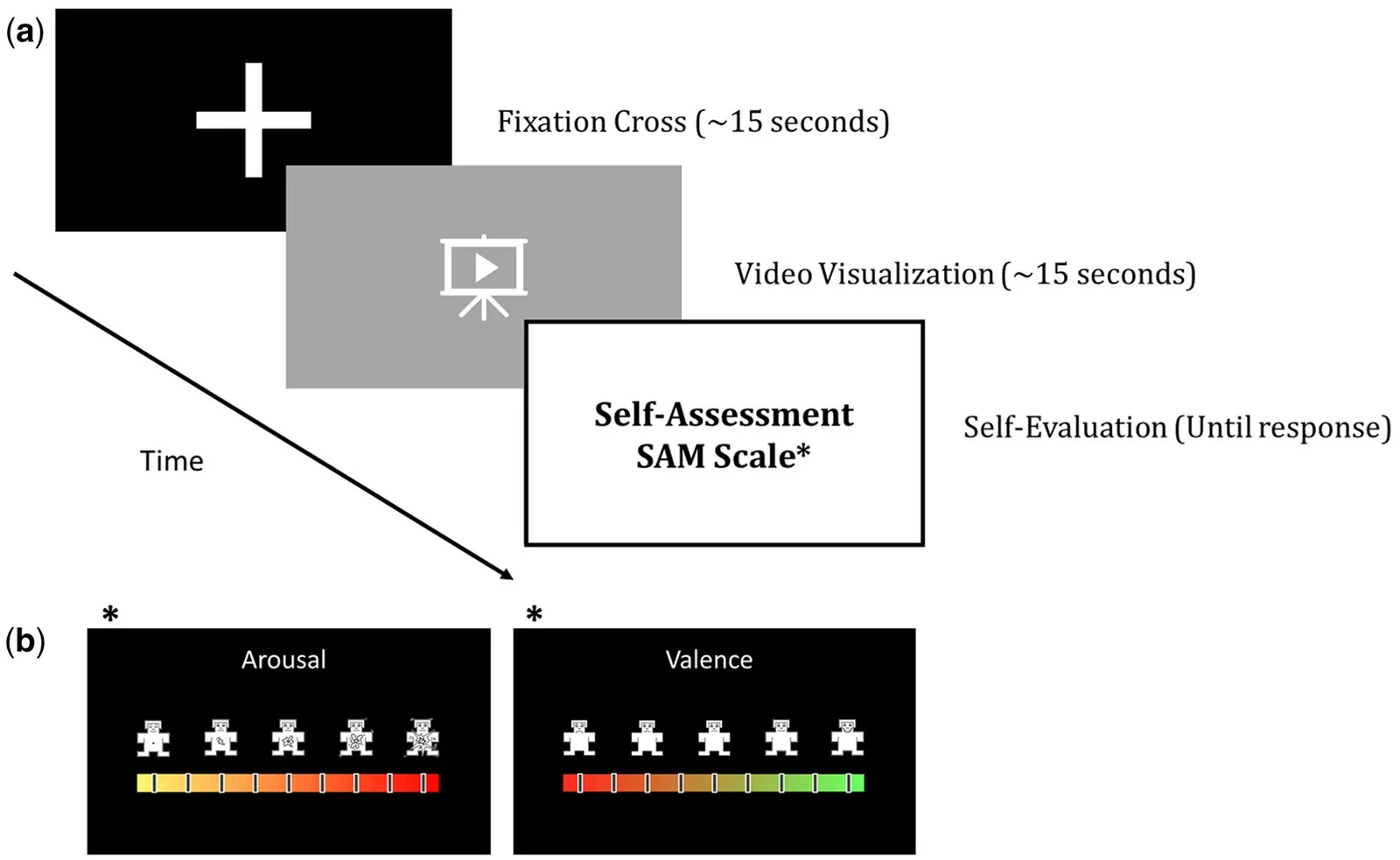
Illustration of what entitles one trial of the fMRI task sequence. (a) The trial comprises three main blocks, fixation cross, video visualization, and self-evaluation. (b) The Illustration of the two 9-point Self-Assessment Manikin (SAM) scales used to rate emotional arousal and valence.

The experimental fMRI task conducted in this study followed a block design and was divided into three runs. Each run comprised 10 trials, during each trial the participants viewed 15-s video clips, featuring varying emotional content. Each trial consisted of three sequential blocks: (1) fixation cross, (2) video visualization, and (3) self-evaluation of valence and arousal states (see Fig. 1A). Before the onset of each video, a fixation cross was presented—consisting of a small white cross against a black background—and remained visible for at least 15 s, matching the duration of the subsequent video. This period served to establish a baseline and facilitate signal normalization. The videos shown during the video visualization block were sourced from the validated CAAV dataset (Di Crosta *et al*. 2020) and each one containing varying levels of emotional arousal and valence. Following the video visualization, participants were prompted to self-evaluate their emotional experiences regarding arousal and valence. This evaluation was conducted using the 9-point Self-Assessment Manikin (SAM) scale (see Fig. 1B).

#### Data acquisition

MRI data were acquired on a 3T Siemens MAGNETOM Prisma Fit scanner (Siemens, Erlangen) equipped with a 64-channel head coil at the Portuguese Brain Imaging Network (Coimbra, Portugal). Functional images were collected using a 2D gradient-echo EPI sequence with simultaneous multi-slice acceleration (factor 6) and in-plane GRAPPA acceleration (factor 2). Acquisition parameters were: TR = 1000 ms, TE = 37 ms, flip angle = 68°, voxel size = 2.0** × **2.0** × **2.0 mm^**3**^, 72 axial slices providing whole-brain coverage, and anterior-posterior phase encoding. To correct for susceptibility-related distortions, we also acquired short spin-echo EPI scans (10 volumes) with reversed phase encoding prior to each run. High-resolution structural images were obtained using a 3D MP2RAGE sequence (TR = 5000 ms, TE = 3.11 ms, 192 interleaved slices, isotropic 1 mm voxels).

#### Data preprocessing

fMRI preprocessing was performed using FMRIB’s Software Library (FSL) (Smith *et al*. 2004, Woolrich *et al*. 2009). Initially, slice timing and motion correction were performed using FSL utility *fsl_slicetimer* and tool MCFLIRT (Jenkinson *et al*. 2002), respectively. Subsequently, a B0-unwarping step was performed with FSL tool TOPUP (Smith *et al*. 2004, Andersson *et al*. 2007), utilizing the reversed-phase encoding acquisition to mitigate EPI distortions. This was followed by bias field correction using the FSL tools FAST (Zhang *et al*. 2001). Following these preprocessing steps, non-brain tissue was removed using FSL tool BET (Smith 2002), rendering the data ready for nuisance regression to remove non-neuronal noise fluctuations (Abreu *et al*. 2017).

Nuisance regression followed the pipeline outlined in (Abreu *et al*. 2017), with nuisance terms regressed out from the fMRI data using a general linear model (GLM) framework. The regressors included in this step were: (1) average BOLD fluctuations measured in white matter (WM) (*r*_WM_) and cerebrospinal fluid (CSF) (*r*_CSF_) masks (obtained as described below), (2) the six motion parameters estimated by MCFLIRT (*r*_MC_), and (3) scan nulling regressors (motion scrubbing) (r_MS_) associated with volumes acquired during periods of significant head motion, identified using the FSL utility *fsl_motion_outliers*. Thus, the final design matrices (*X_i_*) used for nuisance regression were the following:


(1) $$X=[X_{0},\;r_{WM},\;r_{\mathrm{CSF}},\;r_{MC},\;r_{MS}]$$

where, *X*_0_ is a vector of ones. Finally, for both datasets, a high-pass temporal filtering with a cut-off period of 100 s was applied, and spatial smoothing using a Gaussian kernel with full width at half-maximum (FWHM) of 3 mm was performed.

For each participant, WM and CSF masks were obtained from the respective T1-weighted structural images by segmentation into grey matter (GM), WM, and CSF using FSL tool FAST (Zhang *et al*. 2001). The functional images were then co-registered with the respective T1-weighted structural images using FSL’s tool FLIRT, and subsequently with the Montreal Neurological Institute (MNI) (Kasper *et al*. 2017) template, using FSL’s toll FNIRT (Woolrich *et al*. 2009). Both WM and CSF masks were transformed into the functional space and were then eroded using a 3 mm spherical kernel to minimize partial volume effects (Collins *et al*. 1994).

### Statistical analysis

The preprocessed fMRI data were analyzed using a general linear model (GLM) provided by the FSL tool *Feat* at three distinct levels:

#### Participant run-level analysis

Here, each participant’s run was individually analyzed to detect correlates of arousal or valence within each run fMRI blood oxygen level dependent (BOLD) signal at the run level. For this analysis, 3 main effect explanatory variables (EVs) corresponding to different regressors of interest were considered: EV1-Video, corresponding to the segments where the video stimuli were shown, EV2-Arousal, corresponding to the parametric arousal modulation of EV1-Video, and EV3-Valence, corresponding to the parametric valence modulation of EV1-Video.

**Figure 2:**
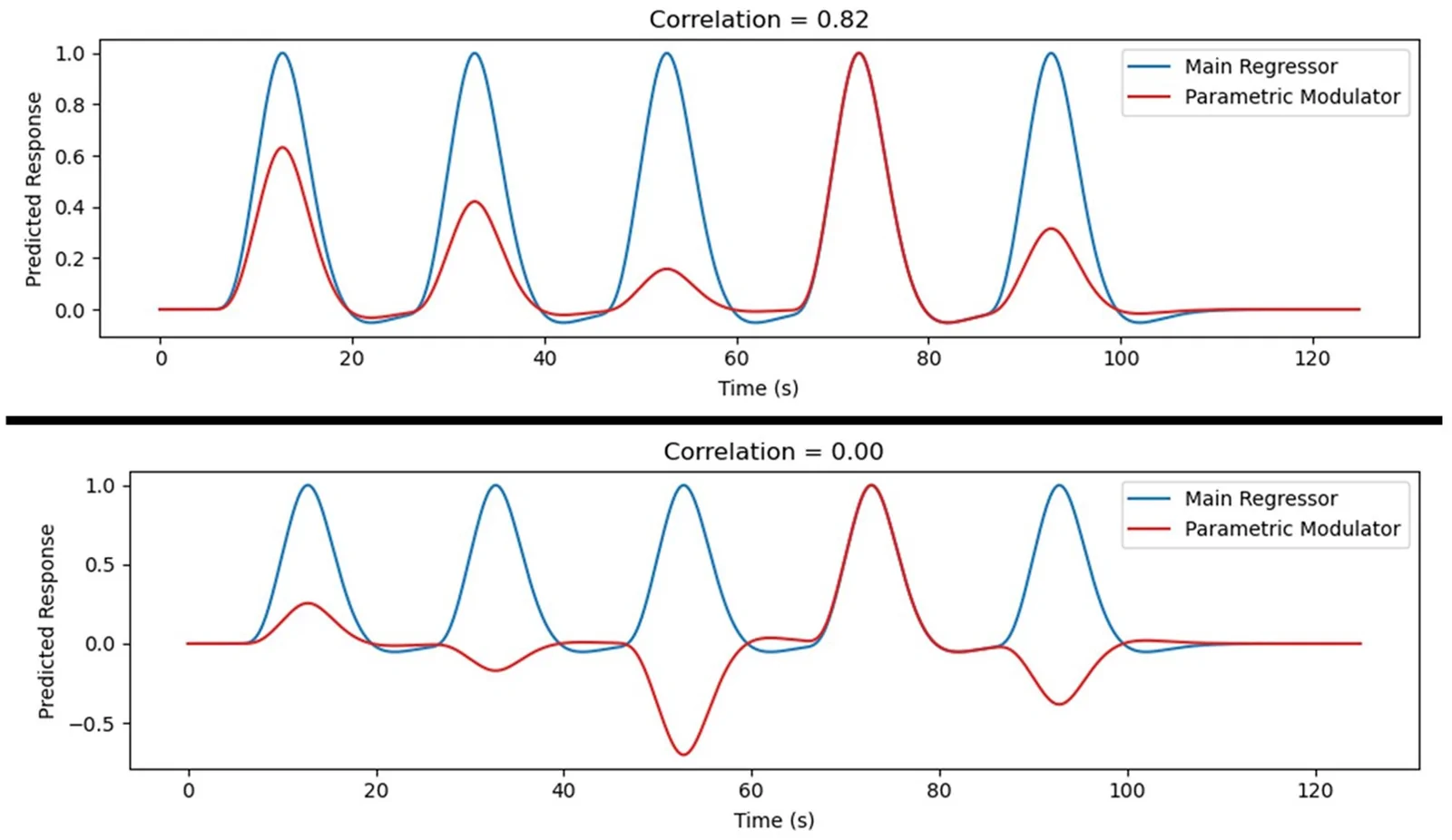
Example of mean centering in the creation of parametric modulators. Before mean-centering, the parametric modulatoris highly correlated with the main regressor (top panel). After mean-centering, the correlation drops to zero (bottom panel).

Each EV was modeled using a boxcar function, containing information about event onset, duration, and intensity. For EV2-Arousal and EV3-Valence, the intensity was modulated by the normalized self-reported arousal and valence ratings for each stimulus. Furthermore, these parametric modulators were made orthogonal to the EV they are modulating (EV1-Video) by mean-centering them (i.e. removing the mean intensity) (Fig. 2). This approach reduces the correlation between the target EV and the modulator EVs, ensuring that each EV explains a unique part of the variance in the fMRI BOLD signal, thereby allowing for more accurate parameter estimation and improved statistical power. Subsequently, each EV was convoluted with the canonical double-gamma Hemodynamic Response Function (HRF), and the BOLD signal from each voxel of the data was fitted accordingly. The fixation-cross segment was intentionally omitted from the model, as Feat considers unmodeled events as baseline, aligning with our intention for this specific event. Additionally, to isolate the effect of emotional arousal and valence during video visualization, the global motion (EV4-Motion) and the presence of humans/human interactions (EV5-Social) in the videos were also modeled as covariates in the final model. For EV4-Motion, the global motion of each video was quantified using motion energy. Motion energy in the context of a video refers to the amount of movement present in the visual content over time. It quantifies the intensity or strength of motion between consecutive frames. The extracted value of motion energy was used to modulate this parametric EV in a similar way previously described for EV2-Arousal and EV3-Valence. The EV5-Social, is a parametric EV that was simply modeled as the EV1-Video, accounting only for the information about event onset, duration, and intensity. For this EV, intensity corresponds to the presence or absence of humans or human interactions in the video. The resulting estimated parameters of each voxel were stored for each EV and utilized for statistical analysis by constructing contrasts, defined in FSL as “Contrasts of Parameter Estimate” (COPE), revealing activation maps related to specific EVs or combinations of EVs. For the first-level analysis, our focus was solely on extracting COPEs related to the parametric modulation of emotional arousal and valence. To ensure a fair modulation and that the final model was not over-specified, we ran our analysis for arousal and valence separately (i.e. with EVs 1-Video, 2-Valence, 4-Motion, and 5-Social for arousal and with Evs 1-Video, 3-Valence, 4-Motion, and 5-Social for valence).

#### Participant-level analysis (second level)

In this analysis, the participant’s average was computed as the average activation for all contrasts of interest across the three runs. A fixed-effect analysis was performed to extract the mean activation of the COPEs of interest across the three runs for each participant, assuming that the effect of the contrasts is consistent within each participant. This approach increases sensitivity by pooling data across runs while controlling for run-level noise, allowing for a more stable estimate of each participant’s brain activation.

#### Group level analysis (third level)

Finally, a statistical group analysis was conducted to summarize the findings across both ASD and TD individuals and to identify group differences. Group activation maps were derived using a mixed-effects analysis, which accounts for both within-subject variability and between-subject variability, allowing for population-level inferences. The resulting z-statistic maps were thresholded at *z* > 3.1 to exclude voxels that were not significantly activated. Additionally, a cluster correction was applied using Gaussian Random Field theory at a significance level of 95%, ensuring that only significant clusters of voxels survived the analysis. At the group level, four COPEs were obtained and used in all subsequent analyses: the parametric modulation of arousal in the TD group, the parametric modulation of arousal in the ASD group, the parametric modulation of valence in the TD group, and the parametric modulation of valence in the ASD group. These COPEs were then used to examine between-group differences in arousal and valence processing.

## Results

### Behavioral data

**Figure 3:**
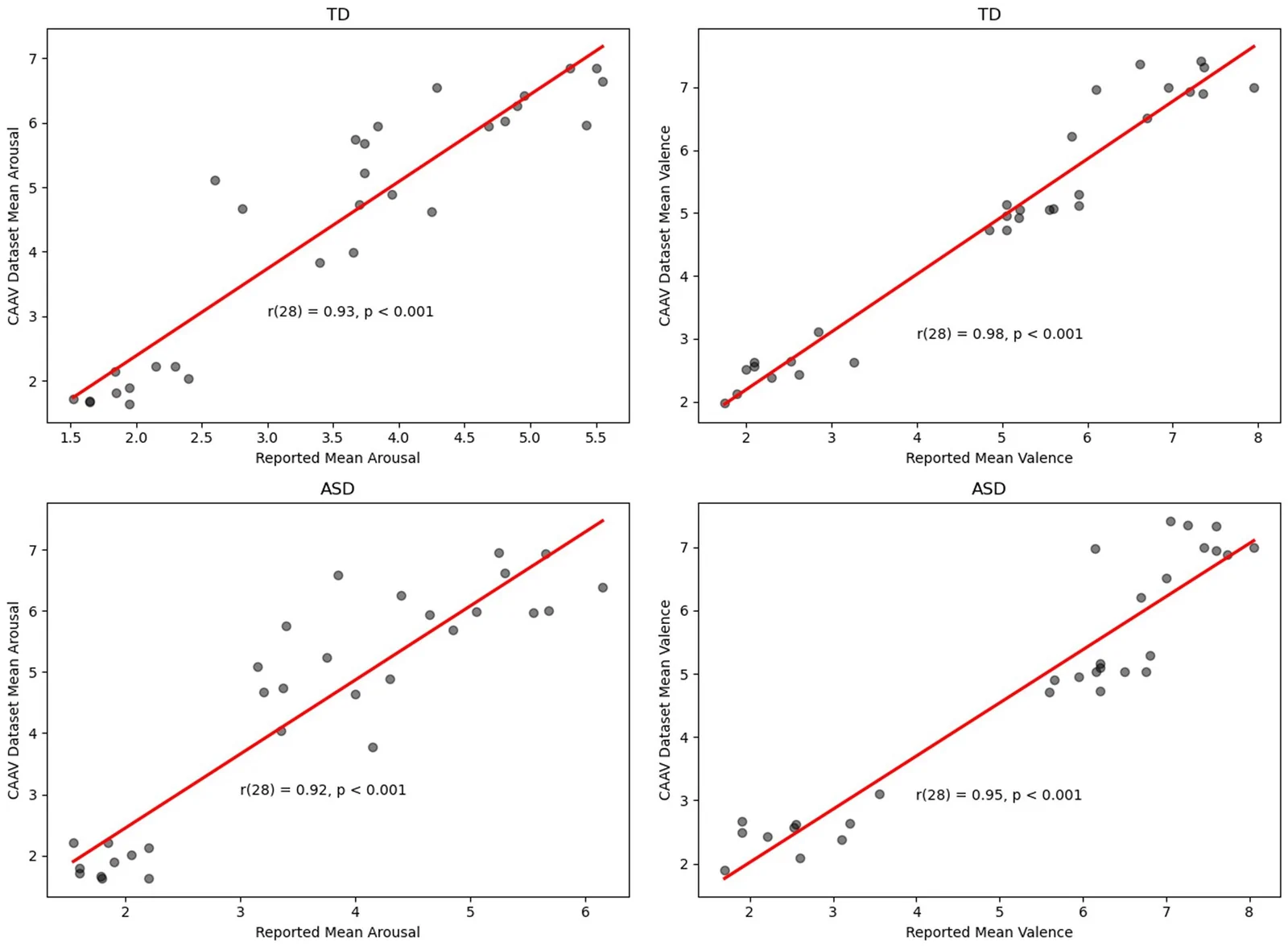
Results from the behavioral analysis. The high Pearson correlation values was used to identify any abnormal ratings of emotional arousal and valence in response to the video stimuli, as well as to validate the selection of the stimuli for both populations.

Behavioral analysis was performed to identify any abnormal ratings of emotional arousal and valence in response to the video stimuli, as well as to validate the selection of the stimuli for both populations. Using Pearson correlation, the results showed that participants from both groups reported levels of arousal and valence for each video, which were highly correlated with the expected arousal and valence levels from the CAAV database (Di Crosta *et al*. 2020) (Fig. 3).

For the typically developing (TD) population, the correlation between self-reported arousal and the expected arousal level was *r*(28) = 0.93, *P* < .001, while the correlation for valence was *r*(28) = 0.98, *P* < .001. For the autism spectrum disorder (ASD) population, the correlation for arousal was *r*(28) = 0.92, *P* < .001, and for valence, *r*(28) = 0.95, *P* < .001.

These high correlation values indicate a strong agreement between participants’ self-reported emotional responses and the standardized emotional levels from the CAAV database (Di Crosta *et al*. 2020). Specifically, both groups rated the emotional arousal and valence of the videos in a manner that closely matched the expected ratings, suggesting that the video stimuli elicited similar perceived emotional responses across populations. This further validates the use of these stimuli to evoke the intended emotional reactions and supports the reliability of participants’ emotional self-evaluations.

### Parametric modulation of emotional arousal

**Figure 4:**
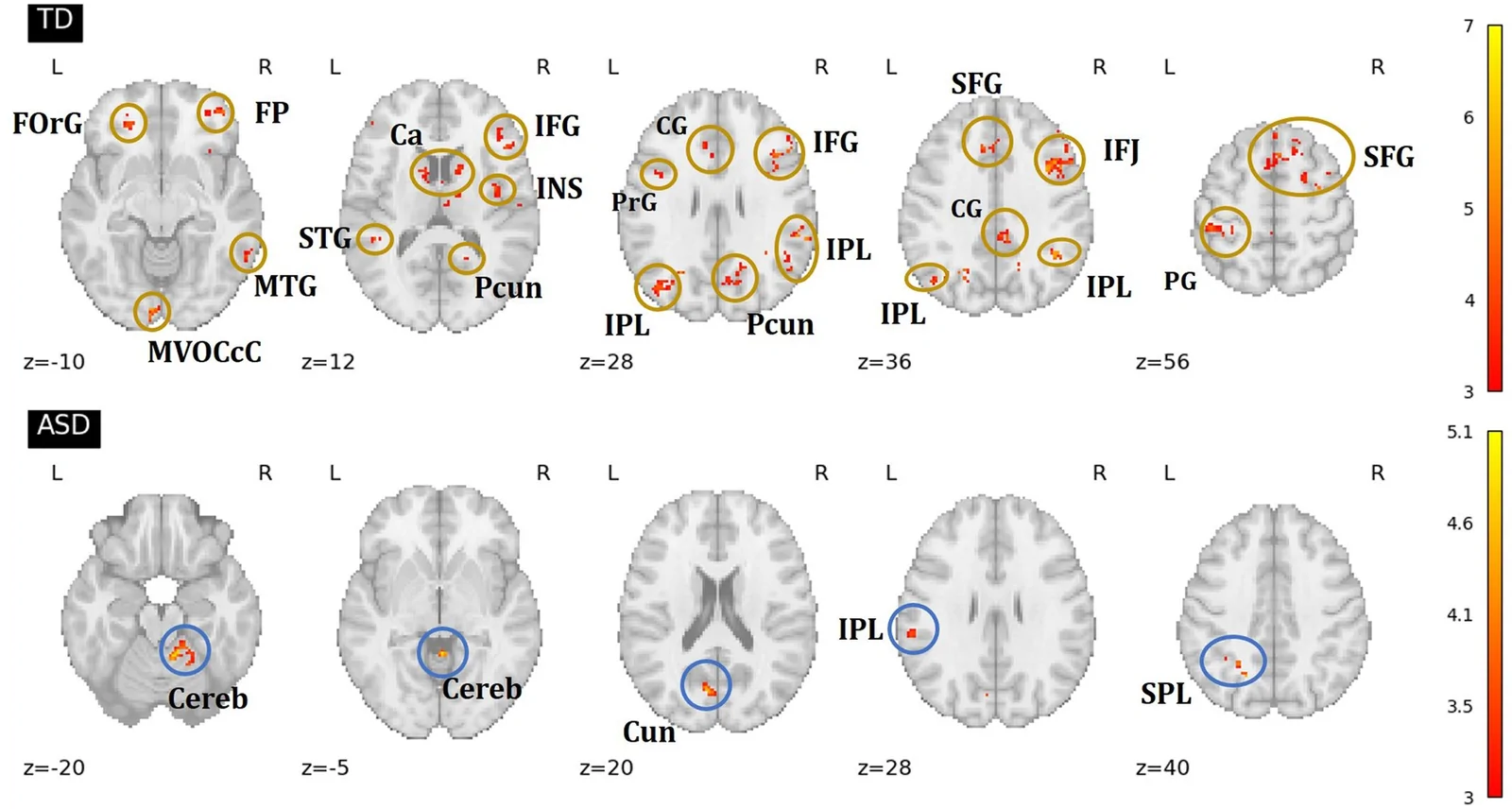
Group-level results from the parametric modulation of emotional arousal. Activation maps depict the brain regions where reported emotional arousal significantly modulated neural activity in a parametric way. The top row presents results for the typical development (TD) group, while the bottom row shows results for the autism spectrum disorder (ASD) group. Statistical maps were thresholded using cluster-based correction (Z > 3.1, *P* < .05, cluster-corrected) as implemented in FSL FEAT, ensuring control over multiple comparisons at a 95% significance level. FOrG = Frontal Orbital Gyrus; FP = Frontal Pole; MVOCcC = MedioVentral Occipital Cortex; MTG = Middle Temporal Gyrus; Ca = Caudate; INS = Insula; STG = Superior Temporal Gyrus; IFG = Inferior Frontal Gyrus; IPL = Inferior Parietal Lobule; Pcun = Precuneus; CG = Cingulate Gyrus; PrG = Precentral Gyrus; SFG = Superior Frontal Gyrus; IFJ = Inferior Frontal Junction; PG = Postcentral Gyrus; SPL = Superior Parietal Lobule.

The results from the parametric modulation of emotional arousal reveal two distinct patterns across the TD and ASD groups (Fig. 4). In the TD group (Table 2), widespread parametric modulation was observed across multiple cortical and subcortical regions. Frontal regions showed strong involvement, including the inferior frontal junction, superior frontal gyrus, middle frontal gyrus, and inferior frontal gyrus. Additional activation appeared in the frontal pole, pars opercularis, and frontal orbital gyrus. In the parietal lobe, significant clusters emerged in the lateral superior occipital cortex, angular gyrus, superior parietal lobule, supramarginal gyrus, and postcentral gyrus, with notable modulation around the temporoparietal junction. Temporal and insular regions also showed significant modulation, including the superior temporal gyrus, middle temporal gyrus, anterior insula, posterior insula, and cingulate gyrus. Subcortical engagement included the thalamus, posterior caudate, and ventral tegmental area. Additional modulation appeared in the precuneus and lingual gyrus.

**Table 2:** Brain regions significantly modulated by emotional arousal in TD.

	Location		MNI coordinates				
Anatomical region	Side	BA	*x*	*y*	*z*	Cluster size	*z*-value
**Frontal lobe**							
** Inferior frontal junction**	R		36	10	36	488	5.78
** Paracingulate gyrus**	L		−8	24	40	146	4.9
** Superior frontal gyrus**	R	2	4	20	56	89	4.8
** Superior frontal gyrus**	R	8	22	−4	50	84	4.64
** Inferior frontal gyrus**	L	39	−40	38	4	63	4.99
** Frontal pole**	R		44	50	−10	56	4.88
** Superior frontal gyrus**	R		14	26	54	49	4.33
** Middle frontal gyrus**	R	37	32	−4	56	44	5.03
** Pars opercularis**	L		−50	18	0	38	4.14
** Superior frontal gyrus**	L	44	−14	34	46	35	4.69
** Frontal orbital gyrus**	L	8	−22	42	−10	34	4.37
** Pars opercularis**	L		−46	8	22	28	4.17
**Parietal lobe**							
** Postcentral gyrus**	L		−46	−30	54	101	4.71
** Precuneus cortex**	R	6	20	−60	26	79	5.45
** Precuneus cortex**	R		4	−38	52	66	5.22
** Angular gyrus**	R	5	52	−62	24	65	5.71
** Supramarginal gyrus**	R	12, 47	56	−38	26	55	5.71
** Angular gyrus**	L	8; 9	−52	−56	42	48	4.81
** Superior parietal lobule**	L	6	−34	−50	50	40	4.45
** Angular gyrus**	L	44	−48	−52	20	27	7.03
** Supramarginal gyrus**	L		−56	−30	44	26	4.69
**Temporal lobe**							
** Lingual gyrus**	L	40	−6	−90	−10	49	5.55
** Superior temporal gyrus**	R	32	50	−18	6	46	4.52
** Middle temporal gyrus**	R	41, 42	58	−50	−6	45	4.56
** Middle temporal gyrus**	L		−64	−48	−4	28	4.5
** Superior temporal gyrus**	L	37	−42	−42	16	28	4.65
**Insular lobe**							
** Posterior insular cortex**	R		40	−4	12	48	4.37
** Cingulate gyrus**	L		−10	26	24	46	4.15
** Anterior insula cortex**	R	39	32	24	−4	27	4.75
**Occipital lobe**							
** Lateral superior occipital cortex**	L		−26	−64	28	201	5.23
**Subcortical**							
** Posterior caudate**	R	5	16	6	16	38	4.12
** Thalamus**	R	11	2	−10	16	33	4.14
** Posterior caudate**	L		−12	−10	20	33	4.31
** Ventral tegmental area**	L		−2	−16	−6	32	3.94
** Posterior caudate**	R		8	2	6	26	4.03

**Figure 5:**
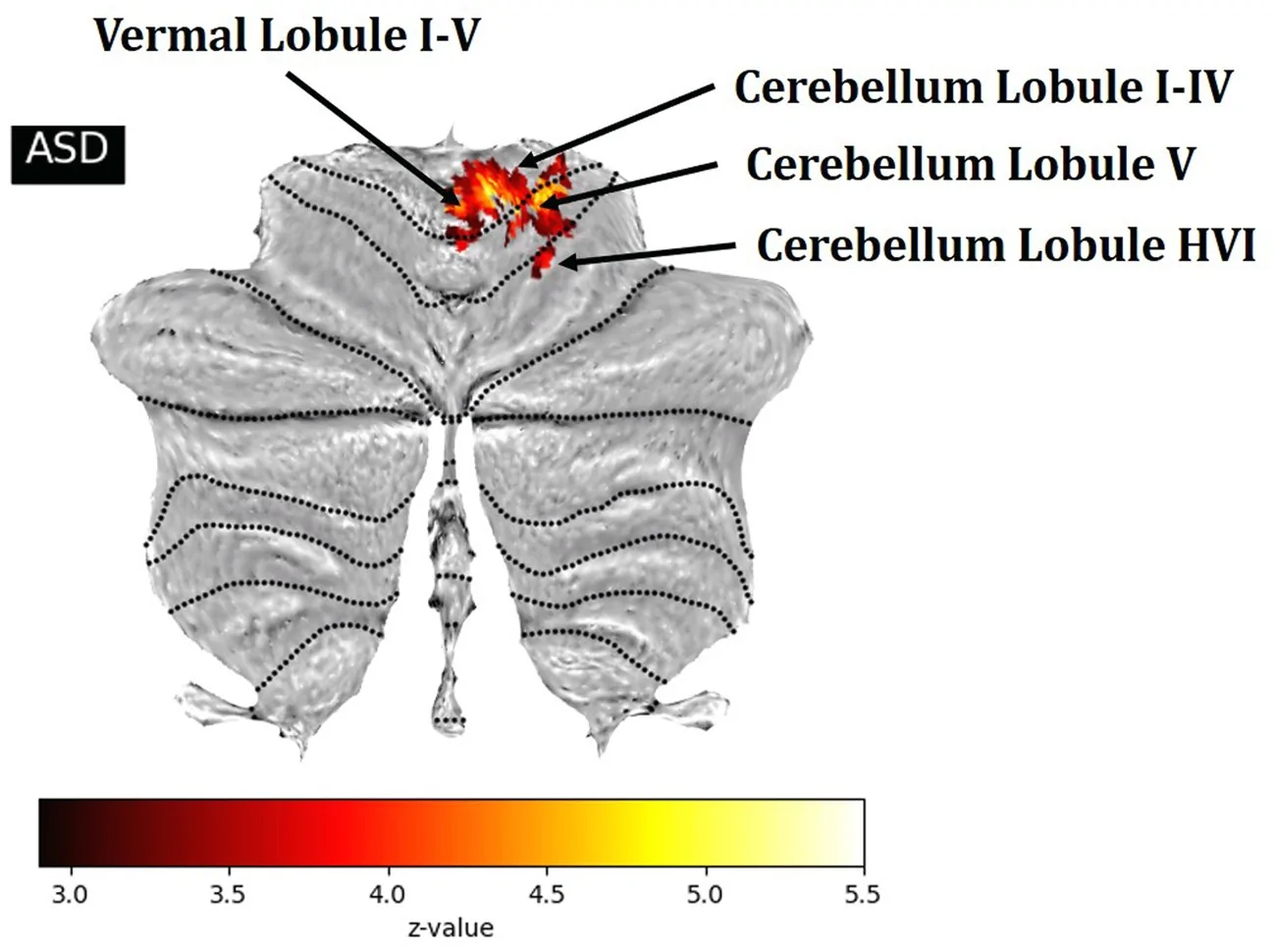
Cerebellar flat map showing the parametric modulation of emotional arousal in the ASD group. Activation is projected onto a flat representation of the cerebellum to facilitate visualization of lobular topology. Warm colors indicate regions where emotional arousal significantly modulated neural activity in a parametric manner. Statistical maps were thresholded at Z > 3.1 (*P* < .05, cluster-corrected) as implemented in FSL FEAT. Notably, significant modulation was observed in the anterior cerebellum, encompassing the vermis and extending into right lobules I–IV and V.

In contrast, the ASD group exhibited a more restricted modulation pattern (Table 3). Significant activation was limited to a single large cluster in the cerebellum that extends from the vermis in cerebellar lobule I–IV to the right lobule I–IV, and lobule V (Fig. 5), in line with the known cerebellar involvement in emotional processing. Within the parietal lobe, modulation appeared in the superior parietal and inferior parietal lobule. Occipital activation was observed in the cuneal cortex, a region associated with visual and affective processing. Compared to TD, the ASD group showed fewer regions with significant modulation (Fig. 4). Notably, frontal and temporal regions strongly engaged in TD showed no significant activation in ASD, indicating divergent neural mechanisms for emotional arousal.

**Table 3:** Brain regions significantly modulated by emotional arousal in ASD.

	Location		MNI coordinates				
Anatomical region	Side	BA	*x*	*y*	*z*	Cluster size	*z*-value
**Cerebellar lobule I–IV and V**	R		20	−38	−24	221	5.13
**Superior parietal lobule**	L	5, 7	−26	−54	38	61	4.92
**Cuneal cortex**	L	17	−8	−72	20	49	4.45
**Inferior parietal lobule**	L	40	60	−31	26	28	3.66

**Figure 6:**
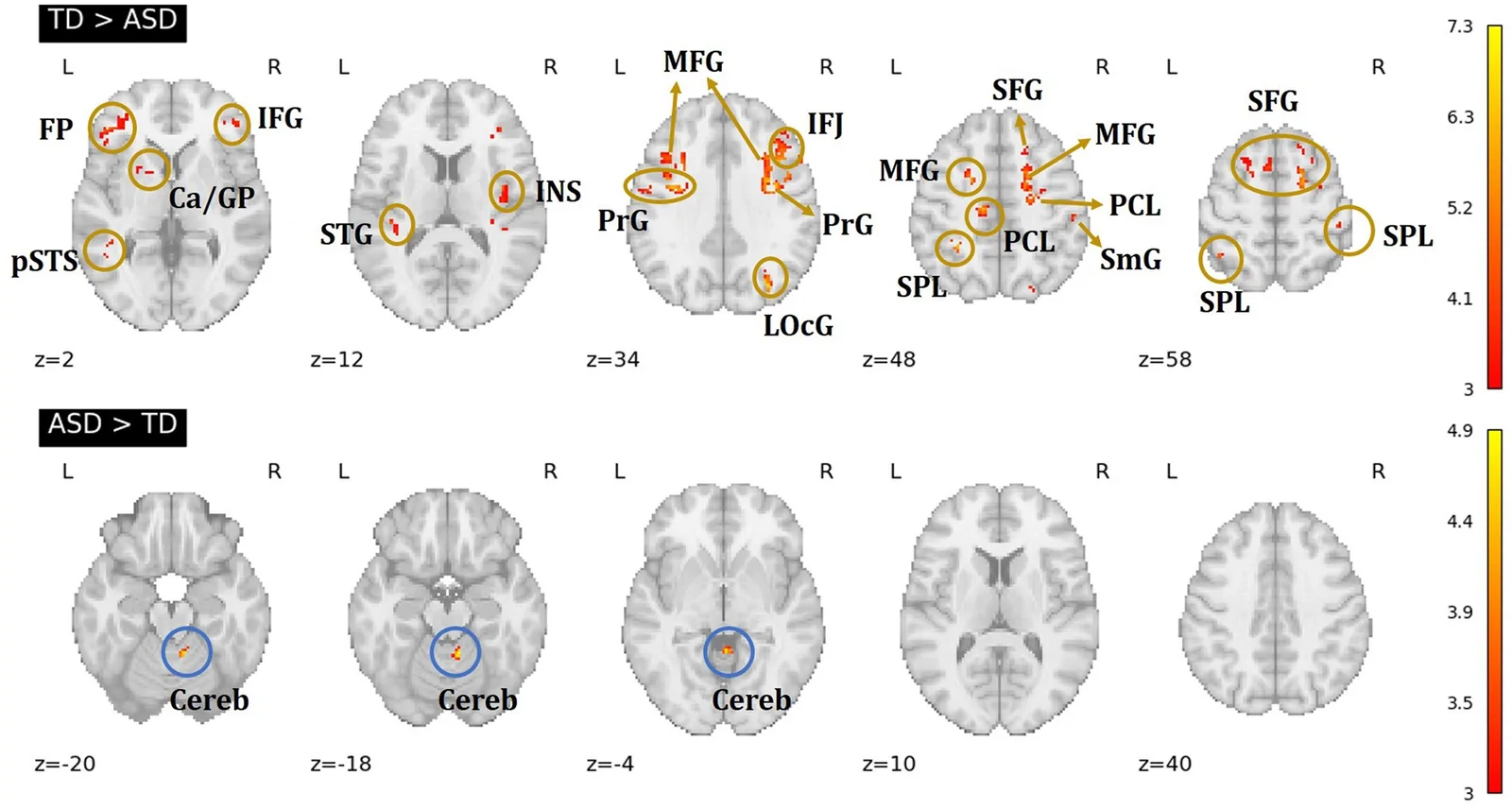
Group differences in the parametric modulation of emotional arousal. Activation maps highlight brain regions where reported emotional arousal significantly modulated neural activity. The top row displays regions with greater activation in the typical development (TD) group compared to the autism spectrum disorder (ASD) group, while the bottom row shows regions with greater activation in the ASD group compared to the TD group. Statistical maps were thresholded using cluster-based correction (Z > 3.1, *P* < .05, cluster-corrected) as implemented in FSL FEAT, controlling for multiple comparisons at a 95% significance level. IFG = Inferior Frontal Gyrus; FP = Frontal Pole; Ca/GP = Caudate and Globus Pallidus; pSTS = posterior Superior Temporal Sulcus; STG = Superior Temporal Gyrus; INS = Insula; MFG = Middle Frontal Gyrus; SFG = Superior Frontal Gyrus; IFJ = Inferior Frontal Junction; PrG = Precentral Gyrus; LOcG = Lateral Occipital Gyrus; PCL = Paracentral Lobule; PcG = Paracentral Gyrus; SmG = Supramarginal Gyrus; SPL = Superior Parietal Lobule.

Direct group comparison (Fig. 6) confirmed these differences. The TD > ASD contrast (Table 4) revealed greater activation in the TD group across frontal regions, including the inferior frontal junction, middle frontal gyrus, superior frontal gyrus, paracingulate gyrus, frontal, and precentral gyrus. These findings reinforce the broader frontal engagement in TD (Table 2), contrasting with ASD (Table 3). Temporal regions also showed greater activation in TD, including the inferior temporal gyrus, middle temporal gyrus, and superior temporal gyrus. The fusiform gyrus was more strongly engaged bilaterally, echoing the within-group differences. TD individuals also showed increased activation in parietal and occipital regions (Table 4), including the superior parietal lobule, angular gyrus, and supramarginal gyrus. Occipital engagement included the lateral occipital cortex, ventromedial parieto-occipital sulcus, and lingual gyrus. Additional differences emerged in subcortical and insular areas, with greater activation in the insular gyrus, posterior insula, globus pallidus, and paracentral lobule, suggesting broader network engagement in TD.

**Table 4:** Brain regions showing significantly greater activation in the TD group compared to the ASD group (TD > ASD) for the parametric modulation of emotional arousal.

	Location		MNI coordinates				
Anatomical region	Side	BA	*x*	*y*	*z*	Cluster size	*z*-value
**Frontal lobe**							
** Inferior frontal junction**	R		32	0	34	789	6.26
** Middle frontal gyrus**	L		−30	14	32	127	5.99
** Middle frontal gyrus**	R	9, 46	32	26	18	115	5.49
** Superior frontal gyrus**	R	8,9	4	22	44	68	4.58
** Precentral gyrus**	L		−34	−6	32	65	6.17
** Superior frontal gyrus**	L	6	−20	2	48	64	5.5
** Frontal pole**	L	45	−44	38	2	59	4.94
** Paracentral lobule**	L	4	−12	−20	46	29	5.97
** Inferior frontal junction**	L		−36	0	38	28	5.35
** Superior frontal gyrus**	L	8	−4	10	58	28	4.19
** Precentral gyrus**	L	4	−54	−6	34	27	4.3
**Parietal lobe**							
** Superior parietal lobule**	L	5	−24	−44	40	116	6.54
** Angular gyrus**	L	39	−36	−54	16	63	6.26
** Supramarginal gyrus**	L	40	−42	−42	24	62	6.41
** Supramarginal gyrus**	R	40	56	−24	46	47	4.68
** Ventromedial parietooccipital sulcus**	L		−24	−70	22	41	7.34
**Temporal lobe**							
** Inferior temporal gyrus**	L		−54	−54	−4	64	5.71
** Inferior temporal gyrus**	R	37	50	−56	−8	46	5.57
** Middle temporal gyrus**	R	21	50	2	−26	40	4.18
** Superior temporal gyrus**	R	41, 42	48	−18	8	26	4.5
**Occipital lobe**							
** Fusiform gyrus**	L	37	−20	−68	−16	177	6.22
** Lingual gyrus**	R		2	−84	−14	109	6.11
** Fusiform Gyrus**	R	37	40	−46	−22	71	5.99
** Lateral occipital gyrus**	R		32	−74	34	70	6.63
** Occipital fusiform gyrus**	R	37	28	−73	−18	57	6.42
** Fusiform gyrus**	L	20	−24	−40	−20	53	5.62
** Lateral occipital gyrus**	R		34	−64	6	30	6.87
** Occipital polar cortex**	L		−60	−94	−4	28	5.18
**Insular lobe**							
** Insular gyrus**	R		36	−26	8	84	4.11
** Posterior insular Cortex**	R		40	−4	12	34	4.79
**Subcortical**							
** Globus pallidus**	L		−20	6	4	26	3.53

**Table 5:** Brain regions with significantly greater activation on the ASD than on the TD group for the parametric modulation of emotional arousal.

	Location		MNI coordinates				
Anatomical region	Side	BA	*X*	*y*	*z*	Cluster size	*z*-value
**Cerebellum right I–IV**	R		8	−48	−18	31	4.87
**Cerebellum right I–IV**	R		2	−46	−7	28	4.82

**Figure 7:**
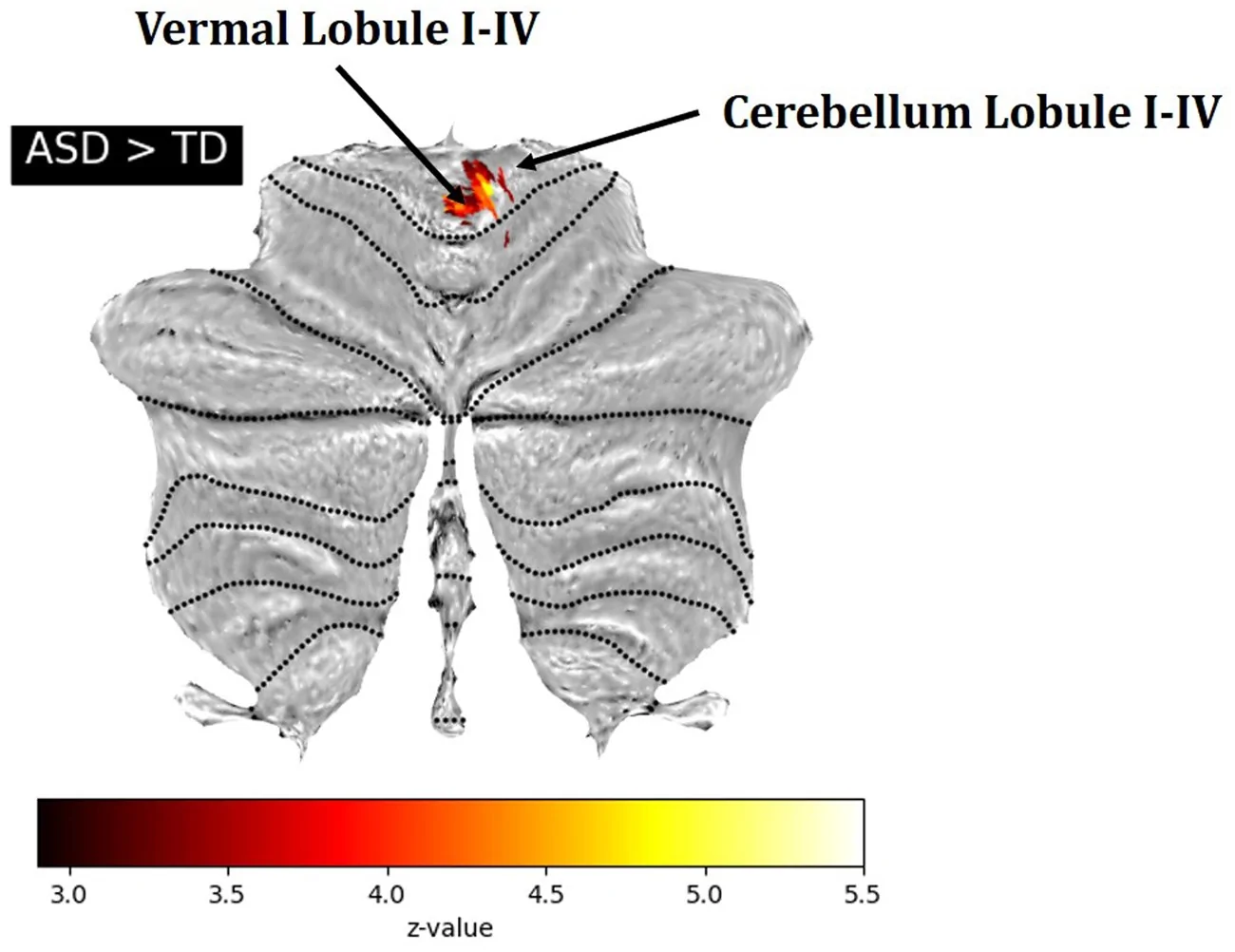
Cerebellar flat map highlighting group differences (ASD > TD) in the parametric modulation of emotional arousal. Clusters represent regions showing greater modulation in ASD relative to TD participants. Statistical thresholding was performed at Z > 3.1 (*P* < .05, cluster-corrected). The main cluster of increased activation was localized in the anterior cerebellum, centered on the vermis and extending to right lobules I–IV, consistent with the within-group ASD findings.

In contrast, the ASD > TD comparison identified only two clusters (Table 5), both in the cerebellum, localized in the vermis and slightly extending towards the right lobules I–IV (Fig. 7). This is consistent with the limited cerebellar involvement seen in the within-group results (Table 3) and may reflect a compensatory arousal related (and not just a sensorimotor difference) in ASD emotional processing.

Overall, the group comparison analysis confirms that the TD group engages a widespread parametric modulation in the cortical network spanning frontal, temporal, parietal, and occipital regions for emotional arousal processing, whereas the ASD group exhibits a markedly different pattern, with limited cortical engagement and increased reliance on the cerebellum. The substantial differences observed in the TD > ASD contrast, combined with the minimal findings in ASD > TD, reinforce the notion that atypical neural mechanisms with much more restricted parametric modulation underlie emotional arousal processing in ASD.

## Discussion

The present study investigated the neural mechanisms underlying the parametric modulation of emotional arousal and valence in individuals with ASD compared to TD controls. The main finding was a striking reduction in arousal-related modulation in ASD, confined primarily to the cerebellum. This result highlights fundamental differences in how individuals with ASD process graded emotional information and supports growing evidence for the cerebellum’s role in affective disturbances and social cognition impairments.

In TD individuals, emotional arousal engaged a widespread cortical and subcortical network, including the cingulate gyrus, insula, caudate, and ventral tegmental area. These structures are consistently implicated in affective processing, emotional regulation, and cognitive control over emotional responses (Šimić *et al*. 2021, Rolls 2023, Zhang *et al*. 2024). In contrast, within the ASD group, parametric modulation was only found in the vermis, mainly subdivisions III and IV, and slightly extending into the lobule I–IV, with an absence of parametric modulation in cortical structures typically involved in emotional processing, suggesting a fundamental difference in how individuals with ASD process grades of emotional arousal.

However, it is noteworthy to point out that the parametric modulation via self-reported arousal may reflect not only core arousal but all the underlying affective, attentional, and social-cognitive processes, underlying the effective introspective integration of emotional stimuli. Although both TD and ASD participants’ ratings of arousal and valence were highly correlated (Fig. 3) with the standardized values of the CAAV database (Di Crosta *et al*. 2020), indicating that both groups perceived the stimuli in a way that closely matched normative expectations, the widespread engagement observed in TD participants may represent the effective integration of multiple layered processes while the lack of cortical engagement found in ASD participant’s might suggest a dissociation between the subjective perception of arousal and its underlying physiological or neural correlates.

Additionally, our findings can also point to differences in the neural implementation of emotional appraisal. Both groups reported emotional experiences consistent with normative expectations; however, they appear to rely on distinct neural strategies, with ASD individuals engaging cerebellar pathways more strongly. The cerebellum’s involvement in ASD has been increasingly documented, extending beyond its traditional role in motor coordination to include contributions to emotion, social cognition, and predictive processing (Courchesne *et al*. 1994, 2007, Siciliano and Clausi 2020).

The cerebellum is organized into ten distinct lobules across three anterior–posterior divisions. The anterior lobe (lobules I–V) is separated from the posterior lobe by the primary fissure, and the posterior lobe (lobules VI–IX) is separated from the flocculonodular lobe (lobule X) by the posterolateral fissure (Schmahmann *et al*. 1999, Stoodley and Schmahmann 2010). Although traditionally viewed as a structure devoted to sensorimotor coordination, a substantial body of work now demonstrates that the cerebellum contributes to a broad range of cognitive, social, and affective functions. Evidence for this expanded role comes from anatomical tracing studies, clinical syndromes following cerebellar damage, and functional neuroimaging (Sacchetti *et al*. 2004, Schmahmann 2004, Sacchetti *et al*. 2007, Adamaszek *et al*. 2017, Apps *et al*. 2018, King *et al*. 2019, Ciapponi *et al*. 2023, Couto-Ovejero *et al*. 2023, Olson *et al*. 2023).

Anatomically, spinal and sensorimotor cortical inputs project primarily to sensorimotor representations located in the anterior lobe (lobules I–V), medial lobule VI, and a secondary somatomotor representation in lobule VIII. This organization is further supported by classical electrophysiological work demonstrating a cerebellar homunculus, an inverted body map, spanning lobules I–V, with a second somatotopic representation bilaterally in lobule VIII (Snider and Eldred 1951). By contrast, intermediate lobules VI–VII are implicated in attentional and executive processes, while posterior regions, notably Crus I, Crus II, and lobules VIII–IX, support higher-order cognitive, social, and affective operations (Stoodley and Schmahmann 2010, 2018). Midline regions, collectively termed the vermis, constitute the cerebellum’s principal interface for integrating sensorimotor, autonomic, and affective signals. These territories project predominantly to the fastigial nucleus, positioning them to modulate arousal, emotional reactivity, and visceral-autonomic responses (Stoodley and Schmahmann 2009, Apps *et al*. 2018, Ciapponi *et al*. 2023). Across species, vermal lobules show distinct functional specializations along the rostro-caudal axis. Rostral vermal lobules IV–VI have been implicated in affective state regulation and fear memory, whereas vermal lobules VI–VII contribute to gaze and oculomotor orientation. More caudally, vermal lobule VIII supports fear-induced freezing and other defensive behaviors, while vermal lobules IX–X are associated with autonomic and cardiorespiratory control. Lesion studies in rodents confirm this functional gradient: damage spanning vermal lobules III–VIII leads to reduced fear responses in anxiogenic environments, diminished freezing to predators, faster recovery from neophobic stimuli, and attenuated predatory behavior, underscoring the vermis as a hub for coordinated emotional-visceromotor processing (Llinás and Wolfe 1977, Supple *et al*. 1987, 1988, Takagi *et al*. 2000, Thier *et al*. 2002, Apps *et al*. 2018).

Furthermore, meta-analysis of fMRI mapping of the human cerebellum provides convergent support for the cerebellum’s involvement in affective processing across both midline and lateral posterior territories. Large-scale quantitative syntheses consistently report emotion‑related activation in vermal lobule VII, lobule VI, lobule IX, Crus I/II, and portions of lobules IV/V, VIIIA/B, and IX, across both hemispheres (Fusar-Poli *et al*. 2009, Stoodley and Schmahmann 2009, E *et al.* 2014, Adamaszek *et al*. 2017, Van Overwalle *et al*. 2024). These findings reinforce that affective processing involves both midline regions (vermis) and posterior cognitive-affective territories, indicating that emotional functions are embedded within broader cerebellar cerebral-cerebellar loops.

As the first implementation of its kind, King *et al*. (2019) provided a major methodological advance by deriving the first fine-grained, within‑subject task-based functional parcellation of the cerebellum. Using a multi‑domain task battery (MDTB) comprising 26 tasks across motor, cognitive, social, and affective domains, they identified 10 functional networks whose boundaries diverged sharply from traditional lobular anatomy. Their parcellation revealed strong vermal recruitment during divided attention, particularly in lobules I–IV, and demonstrated that sad facial expressions reliably activated right lobules IV–VI, Crus I/II, and lobule IX. These findings established a robust functional baseline, demonstrating that cerebellar networks are neither spatially homogeneous nor dominated by motor organization but instead reflect distributed contributions to diverse psychological processes.

Finally, build on all these previous findings, Van Overwalle *et al*. (2024) conducted the most comprehensive large‑scale functional synthesis of the cerebellum to date. Using the NeuroSynth database, they integrated evidence from resting‑state parcellations, task‑based studies, and psychological ontologies to establish a functionally interpretable cerebellar atlas. Their approach employed Activation Likelihood Estimation (ALE) to quantify convergence across thousands of published fMRI studies, resulting in robust activation clusters for 22 psychological domains. Emotion‑related terms yielded consistent activation in vermal lobules I–IV, bilateral lobule VI, and Crus I, with broader emotional constructs showing stronger right‑lateralized engagement in lobule VI and Crus I/II.

Crucially, Van Overwalle *et al*. (2024) used these 22 ALE-derived clusters to re‑evaluate and refine the 10 functional networks originally identified by King *et al*. (2019). To do so, they applied a winner‑take‑all annotation procedure, in which each cerebellar network was assigned the psychological label whose ALE cluster showed the highest feature weight or strongest statistical association with that network’s voxels. In other words, among all 22 psychological domains, the domain that best explained the activation profile of a given network was selected as a descriptor for that network. The resulting functional organization confirmed the major networks identified in earlier resting‑state parcellations, including sensorimotor, directed (dorsal) attention, divided (ventral) attention, and limbic networks (but not the visual network), and showed strong convergence for higher**‑**level associative networks involving executive control, mentalizing (default mode), and language functions. However, similar to prior task‑derived parcellations, the updated atlas revealed reduced symmetry across the two hemispheres.

Accordingly, our results from Figs 5 and 7, revealed a parametric hyperactivation of anterior midline cerebellar regions, specifically around vermal lobules III–IV, with additional extension into the right lobule I–IV in individuals with ASD. Importantly, these territories have been reported in affective paradigms, likely reflecting attentional and sensorimotor‑affective coupling rather than primary emotion specialization, in large‑scale meta‑analyses, which highlight vermal and anterior cerebellar involvement across a variety of affective paradigms (Fusar-Poli *et al*. 2009, Stoodley and Schmahmann 2009, E *et al.* 2014, Adamaszek *et al*. 2017). This, combined with the lack of cortical and subcortical activity might indicate some compensatory mechanism, where ASD individuals, rely less on social cognitive strategies involved in evaluating emotion arousal, and more on basic socio-emotional processing in the cerebellum. In contrast TD participants rely more on wide-spread and multi-processing/domain cortical strategies, consistent with multi‑layered social‑cognitive and interoceptive integration.

Furthermore, converging evidence from King *et al*. (2019) indicates that the same anterior vermal territories (lobule I–IV) are strongly associated with attentional processing, particularly in divided‑attention tasks. This raises the possibility that individuals with ASD may recruit these areas as counterpart to their impaired interoceptive processing and complete access to their bodily arousal level, thus relying more in stimuli queues, which requires higher level of attention, to understand how they are feeling. This interpretation aligns with behavioral evidence that individuals with ASD often struggle to recognize emotions from facial expressions (Law Smith *et al*. 2010, Kliemann *et al*. 2013, Wingenbach *et al*. 2017, Simões *et al*. 2018, Direito *et al*. 2021) and may rely on alternative neural strategies, favoring subcortical or sensorimotor mechanisms rather than the typical cortical networks (Hadjikhani *et al*. 2017). Additionally, this shift in processing strategy aligns with findings on their visual perception abilities, where ASD individuals may overperform in contrast discrimination and detailed analysis of static stimuli but exhibit difficulties with global-motion perception (Chung and Son 2020).

Unlike arousal, the parametric modulation of valence did not reveal significant differences between the groups (Supplementary Figure 1). Both ASD and TD participants engaged similar networks, including frontal and limbic regions such as the superior frontal gyrus, amygdala, and insula, which are widely associated with the evaluation of positive and negative emotional content (Supplementary Tables 1 and 2) (Šimić *et al*. 2021, Zhang *et al*. 2024). These findings, presented in detail in the Supplementary Material, suggest that differences in emotional processing in ASD are specific to the arousal dimension rather than valence. This interpretation is consistent with prior work indicating that ASD-related difficulties often stem from managing the intensity and physiological impact of emotional experiences (i.e. arousal) rather than the categorical appraisal of their positive or negative valence (Mazefsky *et al*. 2013, Mazefsky and White 2014).

Together, these results highlight a dissociation between arousal and valence processing in ASD. Whereas valence appears to be processed in a largely typical fashion, arousal processing is altered, with reduced engagement of cortical regions and increased reliance on cerebellar mechanisms. This distinction is critical, as it underscores the importance of treating arousal and valence as separable dimensions when studying emotional processes in neurodevelopmental conditions. Additionally, the widespread cortical and subcortical activation observed in TD participants resonates with constructivist accounts of emotion, which propose that subjective experiences arise from the integration of multiple psychological processes across distributed networks. Furthermore, our study contributes to this perspective by showing that emotional experiences may be similarly reported across populations, yet underpinned by distinct neural pathways. Overall, these results highlight the need for flexible constructivist perspectives in the study of emotional processing, especially in the context of neurodevelopmental conditions such as ASD.

### Limitations

We would like to acknowledge a limitation of the present study. While self-reported arousal ratings provide valuable insights into subjective emotional experiences, arousal can be more accurately assessed using objective physiological measures, such as heart rate or skin conductance. These measures would allow for a clearer distinction between core arousal signals and broader affective or attentional processes. Despite this limitation, the present findings provide meaningful insights into the neural mechanisms of emotional arousal and valence in ASD and TD individuals. Future studies incorporating objective physiological indices of arousal will be important to validate and extend these results, helping to disentangle core arousal from higher-level cognitive and affective processing and further refine our understanding of emotional modulation in neurodevelopmental populations.

## Conclusion

In this study, our goal was to expand the understanding of how parametric changes in perceived emotional arousal and valence are processed in the brains of TD and ASD individuals as a clue to emotional processing differences between TD and ASD. Our fMRI study provided a fruitful framework for this evaluation, as the video stimuli presented to participants allowed them to experience the scenes as if they were performing the actions themselves, rather than watching static images, creating an immersive experience. Moreover, the CAAV dataset ensured strict control over critical video variables, such as camera angle, number of stimuli, number of actions, and the gender of the actors, making it ideal for isolating the neural correlates of emotional processing.

Behavioral data analysis confirmed that the stimuli used in this study effectively elicited the intended emotional responses in participants. This alignment between self-reported emotions and expected emotional states reinforced the validity of our approach, ensuring that the parametric modulation analysis could reliably capture how these emotional dimensions influence brain activity.

Our parametric modulation paradigm provides valuable insights into the neural mechanisms underlying emotional processing in ASD, by suggesting a critical role for the anterior cerebellum and midline vermis regions. It is well-established that individuals with ASD struggle with recognizing and responding to emotional cues. Our findings support this notion, demonstrating that these differences are primarily driven by the arousal dimension rather than valence. While the TD group engaged a widespread cortical and subcortical network during emotional arousal processing, the ASD group exhibited a markedly reduced parametric modulation pattern, primarily confined to the cerebellum. However, when analyzing emotional valence, both groups showed highly similar activation patterns, with no statistically significant differences in direct comparisons. This suggests that while ASD individuals display atypical neural responses to emotional arousal, their processing of emotional valence remains largely comparable to TD individuals.

## Supplementary Material

nsag026_Supplementary_Data

## Data Availability

Data can be made available upon request.

## References

[nsag026-B1] Abreu R , Nunes S, Leal A et al Physiological noise correction using ECG-derived respiratory signals for enhanced mapping of spontaneous neuronal activity with simultaneous EEG-fMRI. Neuroimage 2017;154:115–27. 10.1016/j.neuroimage.2016.08.008 27530551

[nsag026-B2] Adamaszek M , D’Agata F, Ferrucci R et al Consensus paper: cerebellum and emotion. Cerebellum 2017;16:552–76. 10.1007/s12311-016-0815-8 27485952

[nsag026-B3] Adolphs R , Mlodinow L, Barrett LF. What is an emotion? Curr Biol 2019;29:R1060–R1064. 10.1016/j.cub.2019.09.008 PMC7749626 31639344

[nsag026-B4] Andersson JLR , Jenkinson M, Smith S. Non-linear registration, aka spatial normalization. FMRIB technical report TR07JA2. FMRIB Analysis Group of the University of Oxford; 2007;2:1–22.

[nsag026-B5] Apps R , Hawkes R, Aoki S et al Cerebellar modules and their role as operational cerebellar processing units. Cerebellum 2018;17:654–82. 10.1007/s12311-018-0952-3 PMC6132822 29876802

[nsag026-B6] Baron-Cohen S , Wheelwright S, Skinner R et al The Autism-Spectrum quotient (AQ): evidence from asperger syndrome/high-functioning autism, males and females, scientists and mathematicians. J Autism Dev Disord 2001;31:5–17. 10.1023/a:1005653411471 11439754

[nsag026-B7] Barrett LF. How emotions are made: The secret life of the brain. 2017a.

[nsag026-B8] Barrett LF. The theory of constructed emotion: an active inference account of interoception and categorization. Soc Cogn Affect Neurosci 2017b;12:1–23. 10.1093/scan/nsw154 PMC5390700 27798257

[nsag026-B9] Barrett LF , Satpute AB. Historical pitfalls and new directions in the neuroscience of emotion. Neurosci Lett 2019;693:9–18. 10.1016/j.neulet.2017.07.045 PMC5785564 28756189

[nsag026-B10] Bestelmeyer PEG , Kotz SA, Belin P. Effects of emotional valence and arousal on the voice perception network. Soc Cogn Affect Neurosci 2017;12:1351–8. 10.1093/scan/nsx059 PMC5597854 28449127

[nsag026-B11] Buck TR , Viskochil J, Farley M et al Psychiatric comorbidity and medication use in adults with autism spectrum disorder. J Autism Dev Disord 2014;44:3063–71. 10.1007/s10803-014-2170-2 PMC4355011 24958436

[nsag026-B12] Cabrera M. What is an emotion? Quaderns Filosofia 2021;8:145–91.

[nsag026-B13] Chung S , Son JW. Visual perception in autism spectrum disorder: a review of neuroimaging studies. J Korean Acad Child Adolesc Psychiatry 2020;31:105–20. 10.5765/jkacap.200018 PMC7350544 32665755

[nsag026-B14] Ciapponi C , Li Y, Osorio Becerra DA et al Variations on the theme: focus on cerebellum and emotional processing. Front Syst Neurosci 2023;17:1185752. 10.3389/fnsys.2023.1185752 PMC10206087 37234065

[nsag026-B15] Citron FMM , Gray MA, Critchley HD et al Emotional valence and arousal affect reading in an interactive way: neuroimaging evidence for an approach-withdrawal framework. Neuropsychologia 2014;56:79–89. 10.1016/j.neuropsychologia.2014.01.002 PMC4098114 24440410

[nsag026-B16] Colibazzi T , Posner J, Wang Z et al Neural systems subserving valence and arousal during the experience of induced emotions. Emotion 2010;10:377–89. 10.1037/a0018484 20515226

[nsag026-B17] Collins DL , Neelin P, Peters TM et al Automatic 3D intersubject registration of MR volumetric data in standardized talairach space. J Comput Assist Tomogr 1994;18:192–205. 10.1097/00004728-199403000-00005 8126267

[nsag026-B18] Conner CM , Maddox BB, White SW. Parents’ state and trait anxiety: relationships with anxiety severity and treatment response in adolescents with autism spectrum disorders. J Autism Dev Disord 2013;43:1811–8. 10.1007/s10803-012-1728-0 PMC11097144 23224592

[nsag026-B19] Courchesne E , Pierce K, Schumann CM et al Mapping early brain development in autism. Neuron 2007;56:399–413. 10.1016/j.neuron.2007.10.016 17964254

[nsag026-B20] Courchesne E , Townsend J, Akshoomoff NA et al Impairment in shifting attention in autistic and cerebellar patients. Behav Neurosci 1994;108:848–65. 10.1037//0735-7044.108.5.848 7826509

[nsag026-B21] Couto-Ovejero S , Ye J, Kind PC et al Cerebellar contributions to fear-based emotional processing: relevance to understanding the neural circuits involved in autism. Front Syst Neurosci 2023;17:1229627. 10.3389/fnsys.2023.1229627 PMC10703189 38075533

[nsag026-B22] Dell’Osso L , Massoni L, Battaglini S et al Emotional dysregulation as a part of the autism spectrum continuum: a literature review from late childhood to adulthood. 2023:1–8. 10.3389/fpsyt.2023.1234518 PMC10544895 37791135

[nsag026-B23] Di Crosta A , La Malva P, Manna C et al The chieti affective action videos database, a resource for the study of emotions in psychology. Sci Data 2020;7:32. 10.1038/s41597-020-0366-1 PMC6972777 31964894

[nsag026-B24] Direito B , Mouga S, Sayal A et al Training the social brain: clinical and neural effects of an 8-week real-time functional magnetic resonance imaging neurofeedback phase IIa clinical trial in autism. Autism 2021;25:1746–60. 10.1177/13623613211002052 33765841

[nsag026-B25] E K-H , Chen S-HA, Ho M-HR et al A meta‐analysis of cerebellar contributions to higher cognition from PET and fMRI studies. Hum Brain Mapp 2014;35:593–615. 10.1002/hbm.22194 PMC3866223 23125108

[nsag026-B26] Ekman P. An argument for basic emotions. Cognit Emotion 1992;6:169–200. 10.1080/02699939208411068

[nsag026-B27] Ekman P , Cordaro D. What is meant by calling emotions basic. Emotion Rev 2011;3:364–70. 10.1177/1754073911410740

[nsag026-B28] Fontaine JRJ. Dimensional, basic emotion, and componential approaches to meaning in psychological emotion research. In: Fontaine JRJ, Scherer KR, Soriano C, editors. Components of Emotional Meaning. Oxford (UK). Oxford University Press, 2013, 31–45.

[nsag026-B29] Fusar-Poli P , Placentino A, Carletti F et al Functional atlas of emotional faces processing: a voxel-based meta-analysis of 105 functional magnetic resonance imaging studies. J Psychiatry Neurosci 2009;34:418–32. 10.1139/jpn.0953 PMC2783433 19949718

[nsag026-B30] Gates JA , McNair ML, Richards JK et al Social knowledge & performance in autism: a critical review & recommendations. Clin Child Fam Psychol Rev 2023;26:665–89. 10.1007/s10567-023-00449-0 PMC10613329 37544969

[nsag026-B31] Gotham K , Bishop SL, Hus V et al Exploring the relationship between anxiety and insistence on sameness in autism spectrum disorders. Autism Res 2013;6:33–41. 10.1002/aur.1263 PMC4373663 23258569

[nsag026-B32] Guivarch J , Murdymootoo V, Elissalde SN et al Impact of an implicit social skills training group in children with autism spectrum disorder without intellectual disability: a before-and-after study. PLoS One 2017;12:e0181159. 10.1371/journal.pone.0181159 PMC5513455 28715464

[nsag026-B33] Hadjikhani N , Johnels JÅ, Zürcher NR et al Look me in the eyes: constraining gaze in the eye-region provokes abnormally high subcortical activation in autism. Sci Rep 2017;7:3163. 10.1038/s41598-017-03378-5 PMC5466661 28600558

[nsag026-B34] Hamann S. Mapping discrete and dimensional emotions onto the brain: controversies and consensus. Trends Cogn Sci 2012;16:458–66. 10.1016/j.tics.2012.07.006 22890089

[nsag026-B35] Hirstein W , Iversen P, Ramachandran VS. Autonomic responses of autistic children to people and objects. Proc R Soc B Biol Sci 2001;268:1883–88. 10.1098/rspb.2001.1724 PMC1088823 11564343

[nsag026-B36] Hyde J , Garcia-Rill E. Autism and arousal. In: Garcia-Rill E, editor. Arousal in Neurological and Psychiatric Diseases. Amesterdam: Academic Press. Elsevier, 2019, 83–114.

[nsag026-B37] Jenkinson M , Bannister P, Brady M et al Improved optimization for the robust and accurate linear registration and motion correction of brain images. Neuroimage 2002;17:825–41. 10.1006/nimg.2002.1132 12377157

[nsag026-B38] Kashefimehr B , Kayihan H, Huri M. The effect of sensory integration therapy on occupational performance in children with autism. OTJR 2018;38:75–83. 10.1177/1539449217743456 29281930

[nsag026-B39] Kasper L , Bollmann S, Diaconescu AO et al The PhysIO toolbox for modeling physiological noise in fMRI data. J Neurosci Methods 2017;276:56–72. 10.1016/j.jneumeth.2016.10.019 27832957

[nsag026-B40] Kerns CM , Kendall PC, Berry L et al Traditional and atypical presentations of anxiety in youth with autism spectrum disorder. J Autism Dev Disord 2014;44:2851–61. 10.1007/s10803-014-2141-7 PMC5441227 24902932

[nsag026-B41] King M , Hernandez-Castillo CR, Poldrack RA et al Functional boundaries in the human cerebellum revealed by a multi-domain task battery. Nat Neurosci 2019;22:1371–8. 10.1038/s41593-019-0436-x PMC8312478 31285616

[nsag026-B42] Kinsbourne M. Cerebral-Brainstem relations in infantile autism. In: Schopler, E., Mesibov, G.B., editors. Neurobiological Issues in Autism. Springer, Boston, MA. 1987.

[nsag026-B43] Klein F , Iffland B, Schindler S et al This person is saying bad things about you: the influence of physically and socially threatening context information on the processing of inherently neutral faces. Cogn Affect Behav Neurosci 2015;15:736–48. 10.3758/s13415-015-0361-8 25967930

[nsag026-B44] Kliemann D , Rosenblau G, Bölte S et al Face puzzle-two new video-based tasks for measuring explicit and implicit aspects of facial emotion recognition. Front Psychol 2013;4:376. 10.3389/fpsyg.2013.00376 PMC3693509 23805122

[nsag026-B45] Law Smith MJ , Montagne B, Perrett DI et al Detecting subtle facial emotion recognition deficits in high-functioning autism using dynamic stimuli of varying intensities. Neuropsychologia 2010;48:2777–81. 10.1016/j.neuropsychologia.2010.03.008 20227430

[nsag026-B46] Lindquist KA , Barrett LF. A functional architecture of the human brain: emerging insights from the science of emotion. Trends Cogn Sci 2012;16:533–40. 10.1016/j.tics.2012.09.005 PMC3482298 23036719

[nsag026-B47] Llinás R , Wolfe JW. Functional linkage between the electrical activity in the vermal cerebellar cortex and saccadic eye movements. Exp Brain Res 1977;29:1. 10.1007/BF00236872 408163

[nsag026-B48] Lord C , Risi S, Lambrecht L et al The autism diagnostic observation schedule—generic: a standard measure of social and communication deficits associated with the spectrum of autism. J Autism Dev Disord 2000;30:205–23. 10.1023/A:1005592401947 11055457

[nsag026-B49] Luke L , Clare ICH, Ring H et al Decision-making difficulties experienced by adults with autism spectrum conditions. Autism 2012;16:612–21. 10.1177/1362361311415876 21846664

[nsag026-B50] Mazefsky CA , Herrington J, Siegel M et al The role of emotion regulation in autism spectrum disorder. J Am Acad Child Adolesc Psychiatry 2013;52:679–88. 10.1016/j.jaac.2013.05.006 PMC3719386 23800481

[nsag026-B51] Mazefsky CA , White SW. Emotion regulation. Child Adolesc Psychiatr Clin N Am 2014;23:15–24. 10.1016/j.chc.2013.07.002 PMC3830422 24231164

[nsag026-B52] Mills AS , Tablon-Modica P, Mazefksy CA et al Emotion dysregulation in children with autism: a multimethod investigation of the role of child and parent factors. Res Autism Spectr Disord 2022;91:101911. 10.1016/j.rasd.2021.101911

[nsag026-B53] Muskett A , Radtke S, White S et al Autism spectrum disorder and specific phobia: the role of sensory sensitivity: Brief review. Rev J Autism Dev Disord 2019;6:289–93. 10.1007/s40489-019-00159-w

[nsag026-B54] Olson IR , Hoffman LJ, Jobson KR et al Little brain, little minds: the big role of the cerebellum in social development. Dev Cogn Neurosci 2023;60:101238. 10.1016/j.dcn.2023.101238 PMC10067769 37004475

[nsag026-B55] Orekhova EV , Stroganova TA. Arousal and attention re-orienting in autism spectrum disorders: Evidence from auditory event-related potentials. Front Hum Neurosci 2014;8:34. 10.3389/fnhum.2014.00034 PMC3915101 24567709

[nsag026-B56] Roelofs K , van Peer J, Berretty E et al Hypothalamus-pituitary-adrenal axis hyperresponsiveness is associated with increased social avoidance behavior in social phobia. Biol Psychiatry 2009;65:336–43. 10.1016/j.biopsych.2008.08.022 18947821

[nsag026-B57] Rolls ET. Emotion, motivation, decision-making, the orbitofrontal cortex, anterior cingulate cortex, and the amygdala. Brain Struct Funct 2023;228:1201–57. 10.1007/s00429-023-02644-9 PMC10250292 37178232

[nsag026-B58] Rubin DC , Talarico JM. A comparison of dimensional models of emotion: evidence from emotions, prototypical events, autobiographical memories, and words. Memory 2009;17:802–8. 10.1080/09658210903130764 PMC2784275 19691001

[nsag026-B59] Russell JA. A circumplex model of affect. J Pers Soc Psychol 1980;39:1161–78. 10.1037/h0077714

[nsag026-B60] Sacchetti B , Sacco T, Strata P. Reversible inactivation of amygdala and cerebellum but not perirhinal cortex impairs reactivated fear memories. Eur J Neurosci 2007;25:2875–84. 10.1111/j.1460-9568.2007.05508.x 17466022

[nsag026-B61] Sacchetti B , Scelfo B, Tempia F et al Long-term synaptic changes induced in the cerebellar cortex by fear conditioning. Neuron 2004;42:973–82. 10.1016/j.neuron.2004.05.012 15207241

[nsag026-B62] Schmahmann JD. Disorders of the cerebellum: ataxia, dysmetria of thought, and the cerebellar cognitive affective syndrome. J Neuropsychiatry Clin Neurosci 2004;16:367–78. 10.1176/jnp.16.3.367 15377747

[nsag026-B63] Schmahmann JD , Doyon J, McDonald D et al Three-dimensional MRI atlas of the human cerebellum in proportional stereotaxic space. Neuroimage 1999;10:233–60. 10.1006/nimg.1999.0459 10458940

[nsag026-B64] Scott SK. The acoustic and neurobological bases of the processing of vocal emotion. J Acoust Soc Am 2018;144:1839. 10.1121/1.5068105

[nsag026-B65] Siciliano L , Clausi S. Implicit vs. Explicit emotion processing in autism spectrum disorders: an opinion on the role of the cerebellum. Front Psychol 2020;11:96. 10.3389/fpsyg.2020.00096 PMC7005590 32082228

[nsag026-B66] Šimić G , Tkalčić M, Vukić V et al Understanding emotions: origins and roles of the amygdala. Biomolecules 2021;11:823. 10.3390/biom11060823 PMC8228195 34072960

[nsag026-B67] Simões M , Monteiro R, Andrade J et al A novel biomarker of compensatory recruitment of face emotional imagery networks in autism spectrum disorder. Front Neurosci 2018;12:1–15. 10.3389/fnins.2018.00791 PMC6221955 30443204

[nsag026-B68] Smith SM. Fast robust automated brain extraction. Hum Brain Mapp 2002;17:143–55. 10.1002/hbm.10062 PMC6871816 12391568

[nsag026-B69] Smith SM , Jenkinson M, Woolrich MW et al Advances in functional and structural MR image analysis and implementation as FSL. Neuroimage 2004;23 Suppl 1:S208–19. 10.1016/j.neuroimage.2004.07.051 15501092

[nsag026-B70] Snider R , Eldred E. Electro‐anatomical studies on cerebro‐cerebellar connections in the cat. J Comp Neurol 1951;95:1–16. 10.1002/cne.900950102 14873815

[nsag026-B71] South M , Rodgers J. Sensory, emotional and cognitive contributions to anxiety in autism spectrum disorders. Front Hum Neurosci 2017;11:20. 10.3389/fnhum.2017.00020 PMC5258728 28174531

[nsag026-B72] Stoodley C , Schmahmann J. Functional topography in the human cerebellum: a meta-analysis of neuroimaging studies. Neuroimage 2009;44:489–501. 10.1016/j.neuroimage.2008.08.039 18835452

[nsag026-B73] Stoodley C , Schmahmann JD. Functional topography of the human cerebellum. Handb Clin Neurol 2018;154:59–70. 10.1016/B978-0-444-63956-1.00004-7 29903452

[nsag026-B74] Stoodley CJ , Schmahmann JD. Evidence for topographic organization in the cerebellum of motor control versus cognitive and affective processing. Cortex 2010;46:831–44. 10.1016/j.cortex.2009.11.008 PMC2873095 20152963

[nsag026-B75] Supple WF , Cranney J, Leaton RN. Effects of lesions of the cerebellar vermis on VMH lesion-induced hyperdefensiveness, spontaneous mouse killing, and freezing in rats. Physiol Behav 1988;42:145–53. 10.1016/0031-9384(88)90290-9 3368533

[nsag026-B76] Supple WF , Leaton RN, Fanselow MS. Effects of cerebellar vermal lesions on species-specific fear responses, neophobia, and taste-aversion learning in rats. Physiol Behav 1987;39:579–86. 10.1016/0031-9384(87)90156-9 3588702

[nsag026-B77] Takagi M , Zee DS, Tamargo RJ. Effects of lesions of the oculomotor cerebellar vermis on eye movements in primate: smooth pursuit. J Neurophysiol 2000;83:2047–62. 10.1152/jn.2000.83.4.2047 10758115

[nsag026-B78] Thier P , Dicke PW, Haas R et al The role of the oculomotor vermis in the control of saccadic eye movements. Ann N Y Acad Sci 2002;978:50–62. 10.1111/j.1749-6632.2002.tb07555.x 12582041

[nsag026-B79] Tseng A , Wang Z, Huo Y et al Differences in neural activity when processing emotional arousal and valence in autism spectrum disorders. Hum Brain Mapp 2016;37:443–61. 10.1002/hbm.23041 PMC4734135 26526072

[nsag026-B80] Uljarevic M , Hamilton A. Recognition of emotions in autism: a formal meta-analysis. J Autism Dev Disord 2013;43:1517–26. 10.1007/s10803-012-1695-5 23114566

[nsag026-B81] Van Overwalle F , Ma Q, Haihambo N et al A functional atlas of the cerebellum based on NeuroSynth task coordinates. Cerebellum 2024;23:993–1012. 10.1007/s12311-023-01596-4 PMC11102394 37608227

[nsag026-B82] Velthorst E , Levine SZ, Henquet C et al To cut a short test even shorter: reliability and validity of a brief assessment of intellectual ability in schizophrenia—a control-case family study. Cogn Neuropsychiatry 2013;18:574–93. 10.1080/13546805.2012.731390 23167265

[nsag026-B83] Wingenbach TSH , Ashwin C, Brosnan M. Diminished sensitivity and specificity at recognising facial emotional expressions of varying intensity underlie emotion-specific recognition deficits in autism spectrum disorders. Res Autism Spectr Disord 2017;34:52–61. 10.1016/j.rasd.2016.11.003

[nsag026-B84] Woolrich MW , Jbabdi S, Patenaude B et al Bayesian analysis of neuroimaging data in FSL. Neuroimage 2009;45:S173–86. 10.1016/j.neuroimage.2008.10.055 19059349

[nsag026-B85] Wright B , Kingsley E, Cooper C et al Play brick therapy to aid the social skills of children and young people with autism spectrum disorder: the I-SOCIALISE cluster RCT. Public Health Res 2023;11. 10.3310/VGTR7431 38095124

[nsag026-B86] Zhang R , Deng H, Xiao X. The insular cortex: an interface between sensation, emotion and cognition. Neurosci Bull 2024;40:1763–73. 10.1007/s12264-024-01211-4 PMC11607240 38722464

[nsag026-B87] Zhang Y , Brady M, Smith S. Segmentation of brain MR images through a hidden markov random field model and the expectation-maximization algorithm. IEEE Trans Med Imaging 2001;20:45–57. 10.1109/42.906424 11293691

