# Supplementary material for: Restricted neural parametric modulation of emotional arousal in autism reveals a core role for the cerebellum
Source: Soc Cogn Affect Neurosci. 2026 Apr 9;21(1):nsag026. doi: 10.1093/scan/nsag026 (PMC13221250; doi:10.1093/scan/nsag026)
Supplement: nsag026_Supplementary_Data [file nsag026_supplementary_data.docx]

Restricted Neural Parametric modulation of emotional Arousal in Autism reveals a core role for the cerebellum


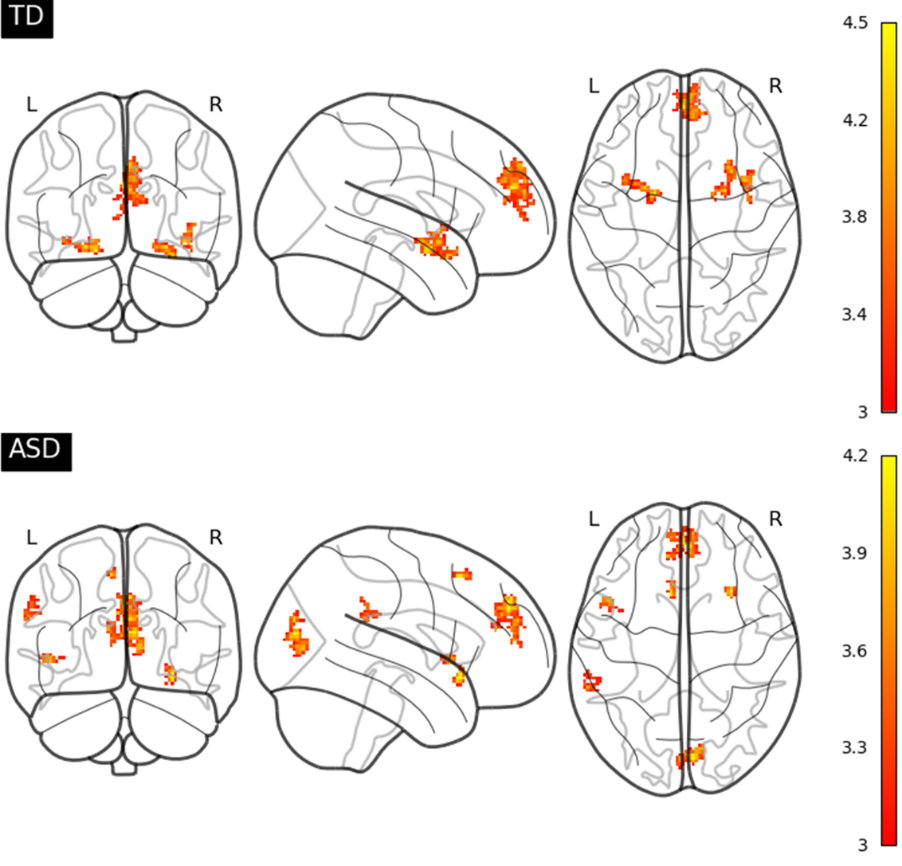


Figure S1. Group level results from the parametric modulation of emotional valence. Activation maps depict the brain regions where reported emotional valence significantly modulated neural activity. The top row presents results for the typical development (TD) group, while the bottom row shows results for the autism spectrum disorder (ASD) group. Statistical maps were thresholded using cluster-based correction (Z > 3.1, p < 0.05, cluster-corrected) as implemented in FSL FEAT, ensuring control over multiple comparisons at a 95% significance level.

Table S1. Brain regions significantly modulated by emotional valence in TD.

|  | **Location** | | **MNI coordinates** | | |  |  |
| --- | --- | --- | --- | --- | --- | --- | --- |
| **Anatomical region** | **Side** | **BA** | ***x*** | ***y*** | ***z*** | **Cluster Size** | **z-value** |
| Superior Frontal Gyrus | L | 10 | 0 | 52 | 24 | 297 | 4.55 |
| Amygdala | L |  | -18 | -2 | -16 | 91 | 4.39 |
| Amygdala | R |  | 26 | 0 | -14 | 79 | 4.30 |
| Insula | R |  | 38 | 6 | -10 | 68 | 4.14 |

Table S2. Brain regions significantly modulated by emotional valence in ASD.

|  | **Location** | | **MNI coordinates** | | |  |  |
| --- | --- | --- | --- | --- | --- | --- | --- |
| **Anatomical region** | **Side** | **BA** | ***x*** | ***y*** | ***z*** | **Cluster Size** | **z-value** |
| Superior Frontal Gyrus | L | 9, 10 | 0 | 50 | 36 | 174 | 4.17 |
| Cuneus Gyrus | R |  | 6 | -84 | 16 | 158 | 4.19 |
| Inferior Parietal Lobule | L | 40 | -60 | -40 | 26 | 47 | 3.68 |
| Frontal Orbital Gyrus | R | 12,47 | 30 | 20 | -12 | 38 | 4.14 |
| Inferior Frontal Gyrus | L | 44, 45 | -46 | 14 | -2 | 34 | 3.85 |
| Superior Frontal Gyrus | L | 8,9 | -8 | 20 | 52 | 29 | 4.14 |
| Superior Frontal Gyrus | L | 10,9 | -8 | 54 | 12 | 27 | 3.69 |
